# Shale gas activity and increased rates of sexually transmitted infections in Ohio, 2000–2016

**DOI:** 10.1371/journal.pone.0194203

**Published:** 2018-03-23

**Authors:** Nicole C. Deziel, Zoe Humeau, Elise G. Elliott, Joshua L. Warren, Linda M. Niccolai

**Affiliations:** 1 Yale School of Public Health, Department of Environmental Health Sciences, New Haven, CT, United States of America; 2 McGill University, Montreal, Canada; 3 Yale School of Public Health, Department of Biostatistics, New Haven, CT, United States of America; 4 Yale School of Public Health, Department of Epidemiology of Microbial Diseases, New Haven, CT, United States of America; Stony Brook University, Graduate Program in Public Health, UNITED STATES

## Abstract

**Background:**

The growing shale gas (“fracking”) industry depends on a mobile workforce, whose influx could have social impacts on host communities. Sexually transmitted infections (STIs) can increase through sexual mixing patterns associated with labor migration. No prior studies have quantified the relationship between shale gas activity and rates of three reportable STIs: chlamydia, gonorrhea, and syphilis.

**Methods:**

We conducted a longitudinal, ecologic study from 2000–2016 in Ohio, situated in a prolific shale gas region in the United States (US). Data on reported cases of chlamydia, gonorrhea, and syphilis by county and year were obtained from the Ohio Department of Health. All 88 counties were classified as none, low, and high shale gas activity in each year, using data from the Ohio Department of Natural Resources. Annual rate ratios (RR) and 95% confidence intervals (95% CIs) were calculated from mixed-effects Poisson regression models evaluating the relationship between shale gas activity and reported annual STI rates while adjusting for secular trends and potential confounders obtained from the US Census.

**Results:**

Compared to counties with no shale gas activity, counties with high activity had 21% (RR = 1.21; 95%CI = 1.08–1.36) increased rates of chlamydia and 19% (RR = 1.27; 95%CI 0.98–1.44) increased rates of gonorrhea, respectively. No association was observed for syphilis.

**Conclusion:**

This first report of a link between shale gas activity and increased rates of both chlamydia and gonorrhea may inform local policies and community health efforts.

## Introduction

Rising global energy demands coupled with technical advancements in the oil and gas industry have led to the economic feasibility and rapid expansion of shale gas extraction. Shale gas extraction involves hydraulic fracturing, commonly known as “fracking,” a stimulation technique used to expel high volumes of natural gas from deep rock formations. Two of the most prolific resources for shale gas extraction in the United States (US) are the Utica and Marcellus shales, both of which are located in eastern Ohio. Over 2,500 shale gas wells were drilled in Ohio from 2009 through 2017 [1].

Proponents of hydraulic fracturing advocate for natural gas as a cleaner alternative to conventional coal-burning power plants, a means to achieve energy independence, and a useful “bridge fuel” until renewables can feasibly be scaled up [2]. Others have raised concerns about air pollutant emissions, water contamination, induced seismic activity, and adverse health outcomes, and quantitative research into these factors is growing [3, 4]. Some proponents of this technology also contend that expansion of the shale gas industry in various geographic areas could lead to localized socioeconomic benefits, such as job creation (e.g., in food service and hospitality industries and truck driving) and increased revenue in the form of increased spending and mineral royalties [5, 6]. Conversely, others have postulated detrimental social impacts of drilling, such as deterioration of roadway infrastructure [7], rise in traffic accidents [8], disruption of community cohesion [9], increased crime [10, 11], and disproportionate siting of waste facilities in communities of disadvantage [12]. However, quantitative research investigating the social impacts of shale gas development remains quite limited.

Shale gas extraction is associated with large influxes of specialized, trained workers into rural areas to meet the labor demands of the drilling rigs, and commonly involves the formation of “work camps” composed of relatively young male workers [13]. Similar experiences with other migratory labor movements have long been recognized to increase risk for sexually transmitted infections (STIs) including HIV [14]. For example, rural-to-urban migration and migration to work in mines in South Africa [15, 16] and oil exploration in Nigeria [17] have documented associations with STI/HIV risk and prevalence. In the US, Latino migrant farm workers in the northwest and southeast and labor migrants to New Orleans post-Katrina were observed to be at high risk for STIs as well [18–20]. The influx of workers in these situations is thought to increase STI risk because male workers typically do not bring families and thus have opportunities to seek other sex partners, they may live and socialize in communities with masculinized social norms and/or they may have few emotional ties to the local community, and sex work may be more available [21–24]. Currently, there is a paucity of research addressing community-level rates of STIs and the influx of workers in areas of shale gas activity.

STIs remain an important and growing global public health issue. There are approximately 20 million new STI cases occurring in the US each year [25], accounting for an estimated $15.6 billion in health care costs [26]. In the US, reported rates of all three reportable non-viral infections, chlamydia, gonorrhea, and syphilis, have remained steady or increased over the past decade [27]. Many chlamydia and gonorrhea infections are asymptomatic and remain untreated, resulting in the potential for serious health consequences such as chronic pelvic pain and ectopic pregnancies, estimated by the Centers for Disease Control and Prevention to cost the US $742 million annually [28]. In 2015, Ohio ranked 16^th^ in the US for chlamydial infections and 12^th^ in gonorrheal infections, both higher than the national average, though rates of syphilis were comparatively lower (ranked 25^th^) [27].

Because STIs are strongly linked to social determinants, understanding community factors that affect the burden of these infections at the population level is critical [29]. As communities may be impacted by the introduction of shale gas development, the population-level associations with STIs need to be understood in order to identify any increase in prevalence that may require intervention. Therefore, the objective of this ecological study was to examine the association between hydraulic fracturing and STI rates in Ohio to gain insight into the social and health impacts of shale gas activity.

## Methods

### Study design and data sources

We conducted an ecological analysis examining the relationship between shale gas activity and reported STI cases from 2000 through 2016. This study draws upon longitudinal data, incorporating a long baseline period prior to the onset of drilling, which commenced in Ohio in 2009. We examined data for the three non-viral STI that are nationally notifiable: chlamydia, gonorrhea, and syphilis. In Ohio, as in all states, laboratory and/or physician diagnoses of these infections are reported to state health departments with the residential address of the case for aggregation to the county-level. Though this is a passive surveillance system, previous studies have shown that STIs are among the most completely reported diseases to the national surveillance system [30]. Data on each STI, reported overall and separately for males and females, for all 17 years and 88 Ohio counties were obtained from the Ohio Department of Health [31]. The annual cumulative incidence of reported cases in each year and county was estimated per 10,000 population as is subsequently referred to as the “annual rate”. Intercensal annual county-level population count estimates (overall, males, females) for years 2000–2016 were obtained from the US Census Bureau [32]; population density was calculated by dividing population estimates by county area (in miles squared) as measured in 2010 [33].

We obtained time-varying, county-level sociodemographic data from the Census Bureau’s decennial census and the American Community Survey (ACS) [33]. We considered the following covariates that have been linked to STI risk and are potentially related to proximity to shale gas activity and work camps: population density (number of people per square mile), income (median household income in USD per year), race (percent population identifying as white only and percent identifying as black only), ethnicity (percent population identifying as Hispanic), access to health care (percent population with health insurance), educational attainment (percent population with a high school diploma and percent population with a Bachelor’s degree), age (percent population aged 15–29 years), and sex (percent population females). The sources and availability of information on these characteristics varied over our study period [33], requiring assumptions in assigning annual values. Sliding 5-year estimates were available from the ACS starting 2005–2009 (2008–2012 for access to health care). Therefore, we applied values from the 2000 Census to all years from 2000–2004 and assigned the 2005–2009 ACS averages to the corresponding time period of 2005–2009 (we assigned the 2008–2012 ACS averages for access to health care to 2000–2012). For the years 2010–2016 (2013–2016 for access to health care), we assigned the 5-year estimate ending in the given year (e.g., for the year 2016, we assigned the 5-year ACS average from 2012–2016).

Data on the locations and permit issue dates of shale gas wells through December 31, 2016 were obtained from the Ohio Department of Natural Resources [1]. Both Marcellus and Utica Shale wells were included. We focused on permits to capture the earlier phases of development, when more workers are required for surveying the land, constructing access roads and well pads, and drilling and fracturing the wells. Approximately 83% of the permitted wells included in our analysis advanced to drilling and production stages. Shale gas activity was quantified using a categorical variable for the number of shale gas wells with permits issued per county and year: none (0 wells), low (1 to 10 wells), and high (>10 wells). Cut-points indicate the number of new well permits below or above the median among the non-zero values across all years of data. This shale gas activity classification was time-varying in that counties were classified each year depending on the number of wells permitted in each county within the respective year. For instance, in 2000, the shale activity classification for all 88 counties was “none,” because shale gas development had not yet begun. In 2012, the year the greatest number of counties had active new permits, 71 counties had no wells permitted that year (“none”), 10 counties had 1–10 new well permits issued in that year (“low”), and 7 counties had >10 wells permits issued (“high”). We included year as a categorical variable in our model to conservatively control for temporal changes in reported STI rates without making assuming about the shape or properties of the secular trends.

### Statistical analysis

We estimated annual rate ratios (RRs) and 95% confidence intervals (95% CIs) for the relationship between shale gas activity and reported STI cases using generalized linear mixed models in the form of Poisson regressions with random effects. This model is widely used in epidemiological studies to analyze longitudinal data in which the response variable is a disease count occurring over a given time period. In our regression model, the logarithm of county population estimates in each year were included as an offset term. We also included a random effect for county to allow each county to have its own baseline rate and to account for potential correlation in rates across time within a county. We added an observation-level random effect to account for possible overdispersion (excess variability than expected), which is common in count data and, if ignored, can lead to an underestimation of standard errors for the fixed effects of interest. Shale gas activity was included in all models as our main effect of interest. All continuous covariates were standardized prior to running the final model by subtracting the mean and dividing by the standard deviation to provide computational stability. We conducted a Spearman’s correlation analysis among all continuous covariates to determine if multicollinearity was an issue for our modeling. When two covariates were highly correlated (|*r*_*Spearman*_| > 0.7, we only retained one in the model. Based on those results, we excluded the following variables from the final models: (i) percent of the county population that was black (negatively correlated with percent white) and (ii) county-level education measures (positively correlated with several covariates including percent with health insurance, population density, and median household income). The variables selected for inclusion in the final models were shale gas activity, median household income, percent population with health insurance, percent white, percent Hispanic, percent population 15–29 years, percent females, population density, and year. We retained all selected variables in the final models regardless of statistical significance, which was set at 0.05. All regression analyses were conducted using Proc Glimmix in SAS 9.3 (SAS Institute, Cary, NC, USA).

We conducted a residual analysis and created residual plots to visualize the impact of hydraulic fracturing on each of the STI outcomes over time, after accounting for the other STI risk factors. We refit our statistical models including all covariates except the shale activity variable. The residuals from these analyses, defined as observed minus predicted, reflect the additional variability in STI outcomes that could be attributable to shale activity (or other unknown factors) since the effects of the confounding variables (including year) were controlled. We plotted the mean residuals across all counties by year, separately for each shale gas activity categories.

### Sensitivity analyses

We carried out several sensitivity analyses to assess the robustness of our findings for various model assumptions. We reran our models using a cumulative, rather than annual, number of permits as our metric of interest for shale gas activity. We repeated our modeling using year as a continuous, rather than categorical, variable. We also examined the relationships between shale gas and STI rates stratified by sex. Finally, we ran our model using Salmonellosis, a common foodborne illness, as a negative control outcome variable (data obtained from the Ohio Department of Health), because an association between shale gas activity and an infectious disease not sharing the same hypothesized mechanism as that for STIs could indicate the presence of unmeasured confounders [34]. We also included Salmonellosis counts as an independent variable in our STI models in a subsequent sensitivity analysis to potentially account for any unmeasured confounding. In addition, we tested whether the county-level random effects properly accounted for spatial correlation in the data by calculating Geary’s C test statistic for the model residuals and conducting hypothesis tests at each year separately (17 years, 3 STIs; total of 51 hypothesis tests). A Bonferroni correction was used to account for the multiple hypothesis tests being conducted. We expected the residuals to be uncorrelated spatially during each year of the analysis due to the inclusion of the county-level random effects.

## Results

**Fig 1:**
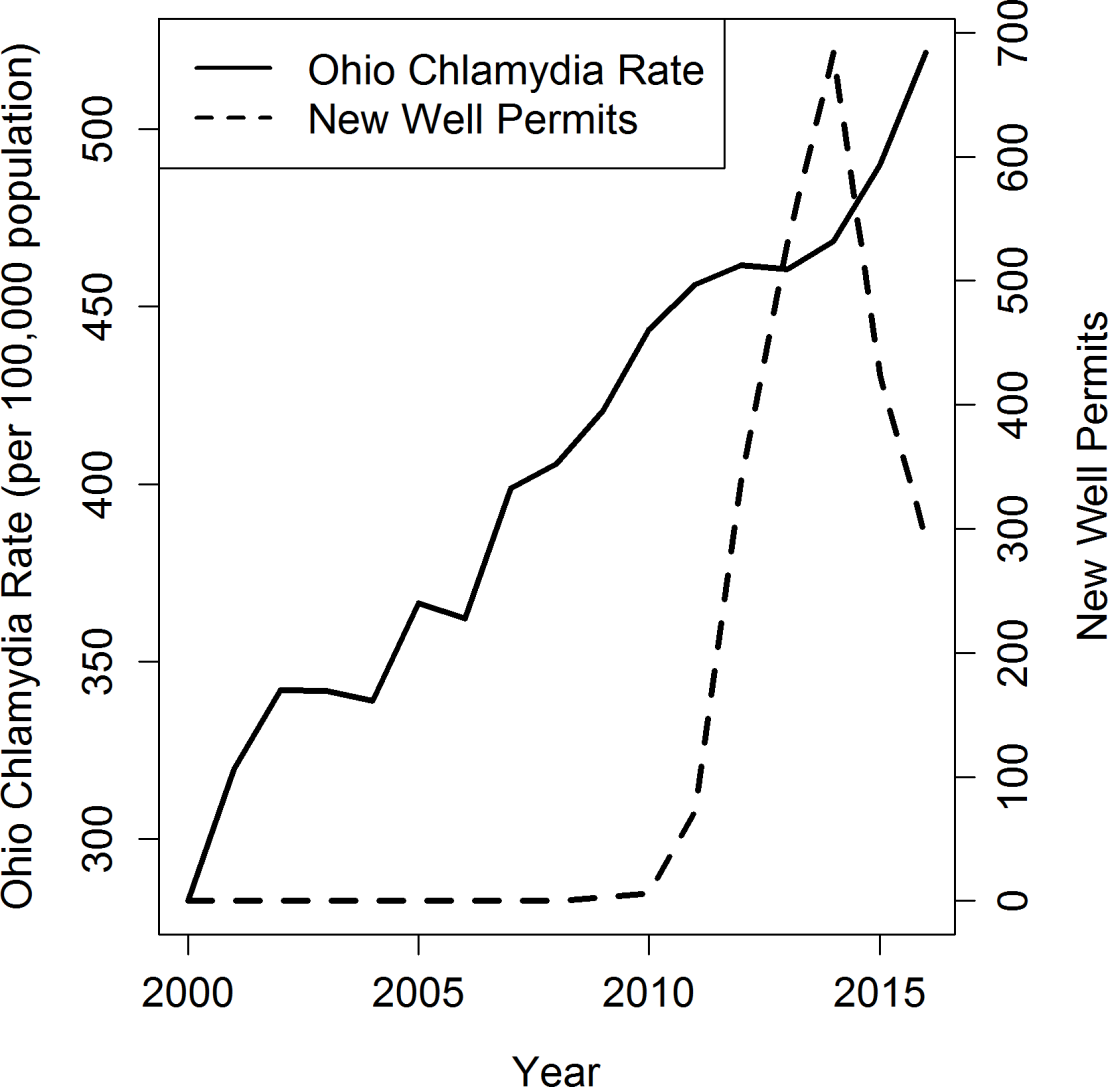
Temporal trends in total reported chlamydia rates and new well permits in Ohio 2000–2016.

**Fig 2:**
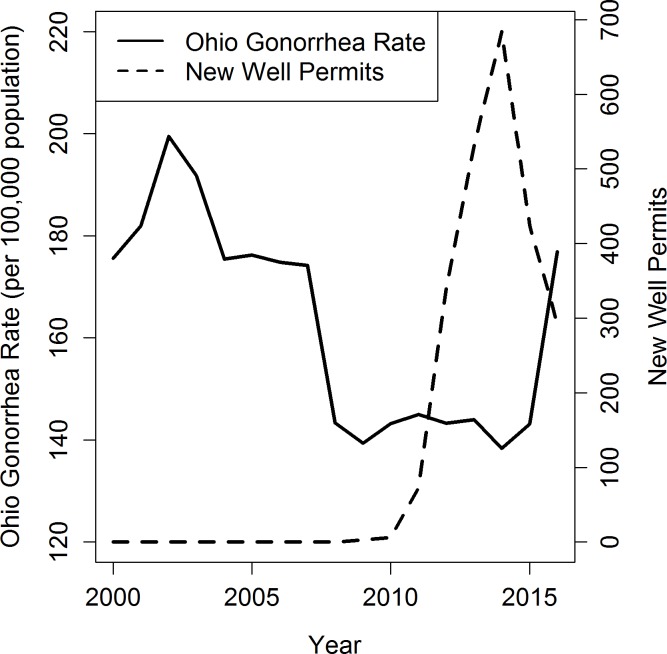
Temporal trends in total reported gonorrhea rates and new well permits in Ohio 2000–2016.

**Fig 3:**
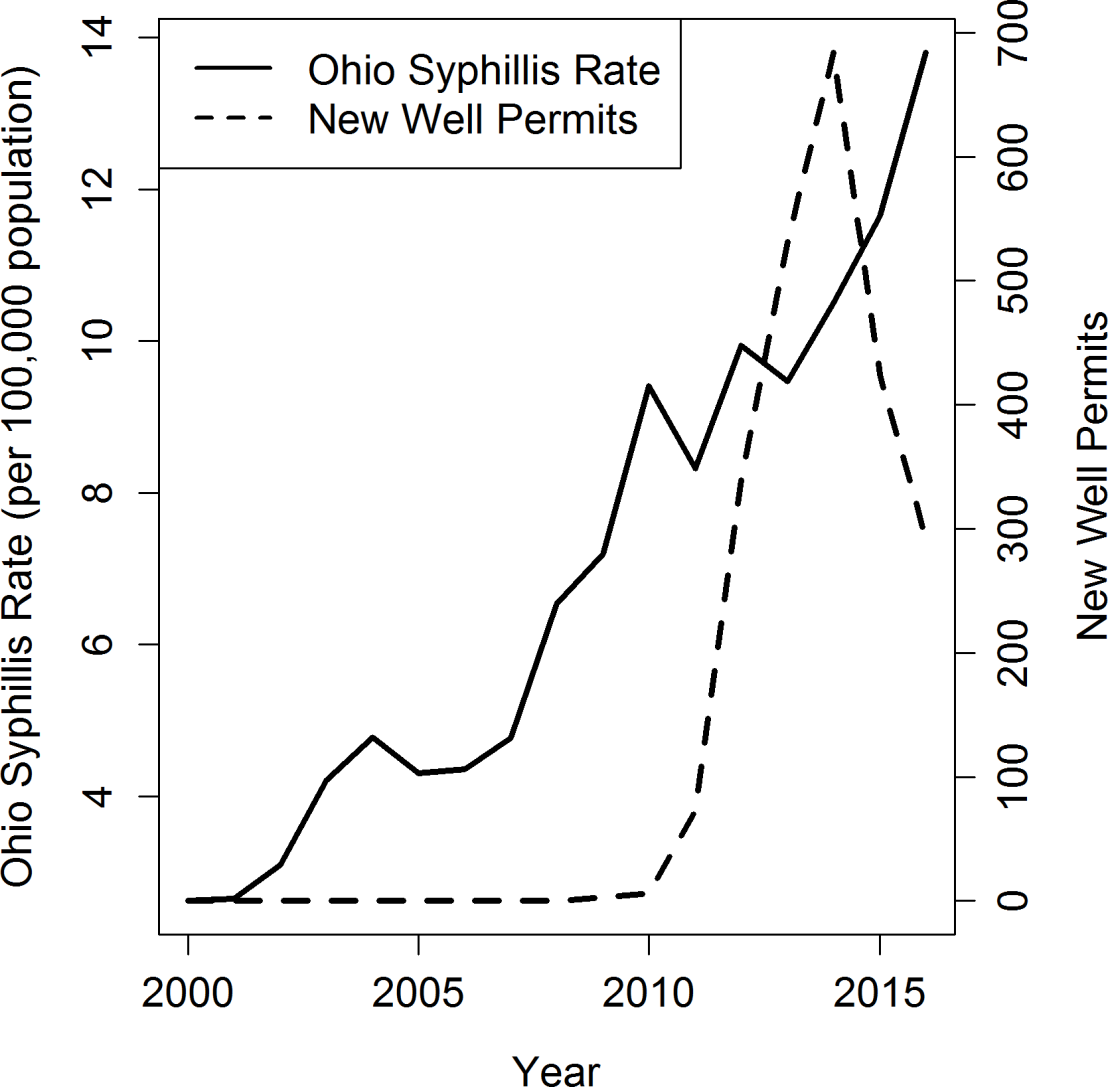
Temporal trends in total reported syphilis rates and new well permits in Ohio 2000–2016.

Shale gas activity began in Ohio in 2009 with three wells and increased rapidly during 2010–2014, followed by a decline in the number of new permits (Figs 1, 2 and 3), mirroring regional trends in Appalachia ([35]). Mean annual rates for chlamydia and syphilis increased steadily over most of the study period, while the mean annual gonorrhea rate declined until 2010, when it began increasing (Figs 1, 2 and 3). Reflecting national patterns, reported rates were highest for chlamydia, followed by gonorrhea and syphilis.

**Fig 4:**
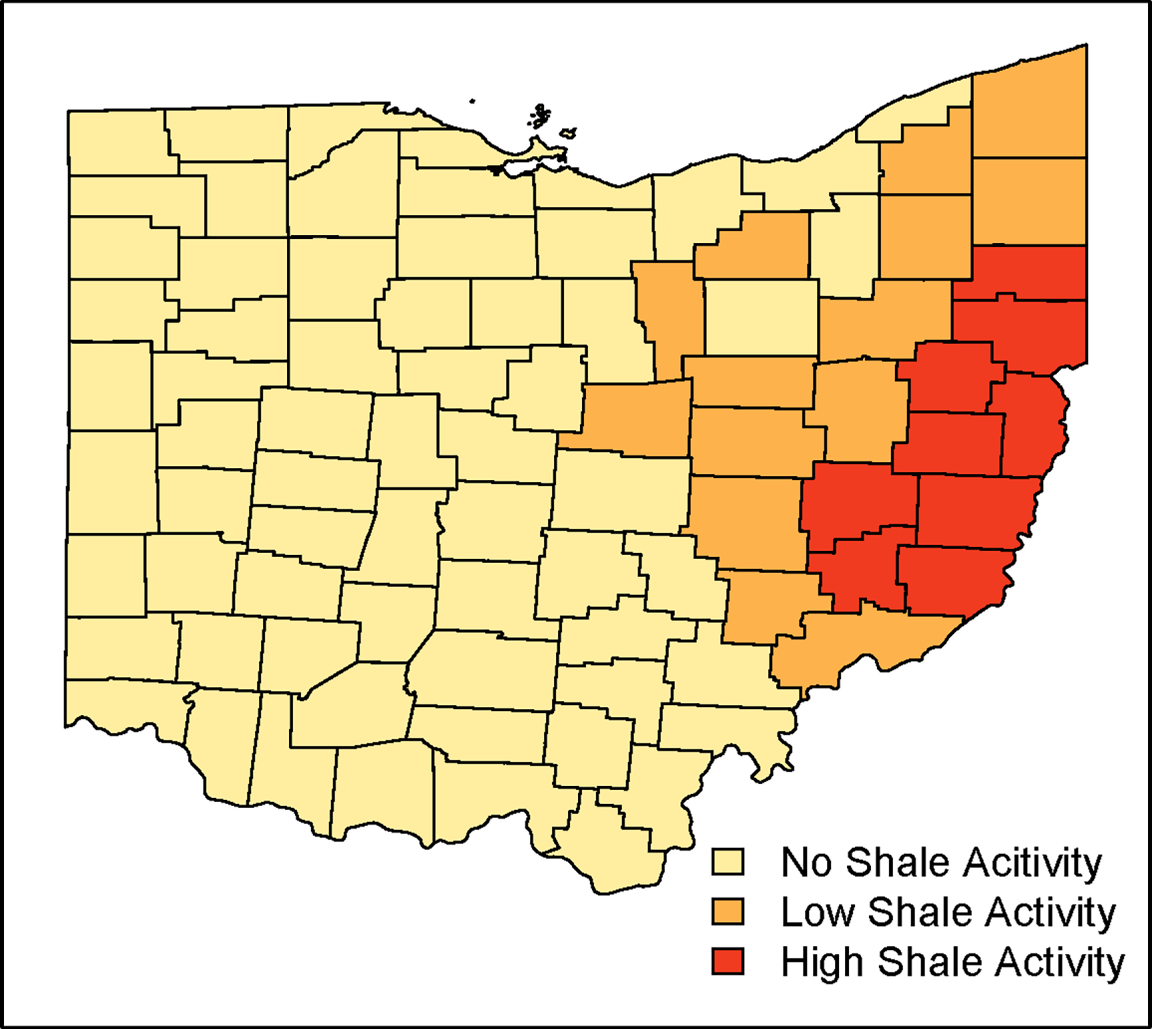
Maximum shale gas activity status reached by Ohio counties.

Counties that ever experienced shale gas activity (n = 23) tended to be less densely populated, have lower income, a greater percentage of people reporting as white, lower percentage of people reporting as Hispanic, and lower percentage of people with health insurance compared to counties without shale activity (n = 65), though only the difference in Hispanic ethnicity was statistically significant (Table 1). Our study period included 1496 county-years (88 counties x 17 years); of these 47 (3.1%) county-years were at the low drilling activity, and 38 county-years (2.5%) reflected the high drilling category. All the counties with shale gas activity were located in eastern Ohio, situated above the Marcellus and Utica Shale formations (Fig 4).

**Table 1:** Distribution of sociodemographic variables among counties ever experiencing any and no shale activity in Ohio from 2000–2016.

Sociodemographic Factor	Ever Any Shale Activity (n = 23)	No Shale Activity (n = 65)	
	Mean (SD)	Mean (SD)	P-value
Population density (people per mile^2^)^a^	180 (168)	268 (410)	0.16
Median household income (USD)	42,100 (7,970)	45,724 (8,487)	0.08
Percent with health insurance	86 (7)	89 (2)	0.10
Percent females	51 (2)	51 (1)	0.91
Percent white	94 (4)	92 (7)	0.08
Percent black	3 (4)	4 (6)	0.36
Percent Hispanic	1 (1)	2 (2)	<0.001
Percent aged 15–29 years old	19 (2)	20 (3)	0.14
Percent with high school diploma	84 (7)	85 (4)	0.38
Percent with Bachelor’s degree	15 (6)	17 (8)	0.22

^a^Statistically significant differences in mean values by shale activity status, based on two-sample t-tests.

IQR, inter-quartile range; SD, standard deviation; USD, United States dollars

Table 2 presents the annual rate ratios for chlamydia, gonorrhea, and syphilis in relation to extent of new shale gas activity (low or high), unadjusted and adjusted for model covariates. We observed elevated ratios for chlamydia and gonorrhea, particularly at the high activity level. Compared to counties without any shale gas development, counties with high activity had 21% (RR = 1.21; 95% CI = 1.08–1.36) increased rate of chlamydia and 19% (1.19; 0.98–1.44) higher rates of gonorrhea, after adjusting for model covariates. In contrast, there was no association between shale gas extraction activity and syphilis rates.

**Table 2:** Rate ratios (RR) and 95% confidence intervals (95% CI) for the association between shale gas activity and reported rates of sexually transmitted infections in Ohio 2000–2016 (n = 1496 county-years).

	County-	Unadjusted^a^	Adjusted^b^
	Years	RR (95% CI)	RR (95% CI)
Chlamydia			
None	1411	1.0	1.0
Low Activity	47	1.50 (1.32, 1.70)	0.99 (0.91, 1.08)
High Activity	38	2.16 (1.85, 2.13)	1.21 (1.08, 1.36)
Gonorrhea			
None	1411	1.0	1.0
Low Activity	47	1.14 (0.99, 1.31)	1.14 (0.98, 1.31)
High Activity	38	1.31 (1.08, 1.58)	1.19 (0.98, 1.44)
Syphilis			
None	1411	1.0	1.0
Low Activity	47	1.21 (0.85, 1.73)	0.86 (0.62, 1.20)
High Activity	38	1.45 (0.87, 2.42)	0.71 (0.44, 1.16)

^a^Model includes log(population) as offset term and county-level random effect.

^b^Adjusted for population density, median household income, % with health insurance, % white, % Hispanic, % Population 15–29 years old, % female, year; model also includes log(population) as offset term and county-level random effect.

**Fig 5:**
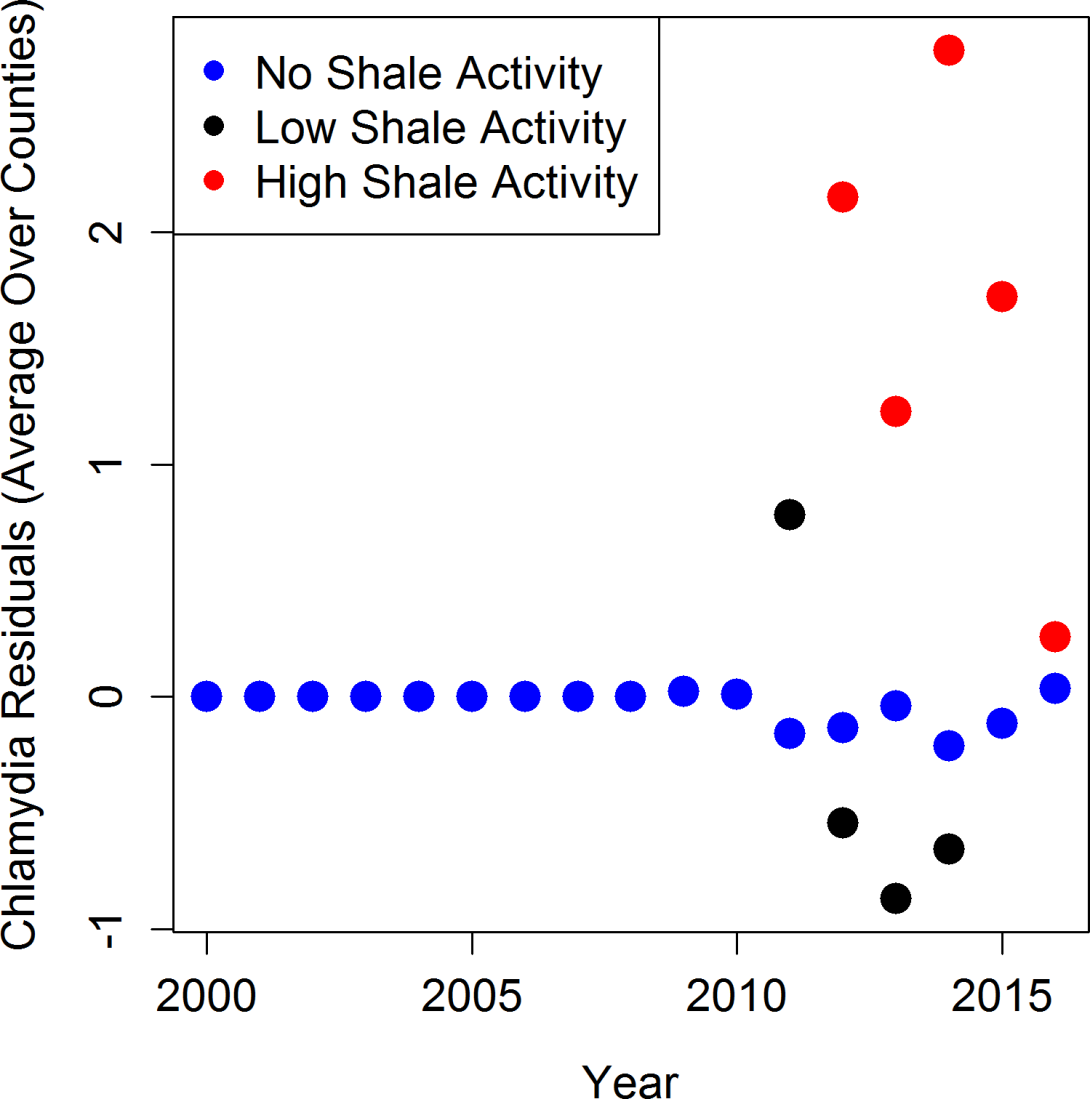
Mean residuals across counties and time by activity categories for chlamydia models excluding activity variable.

**Fig 6:**
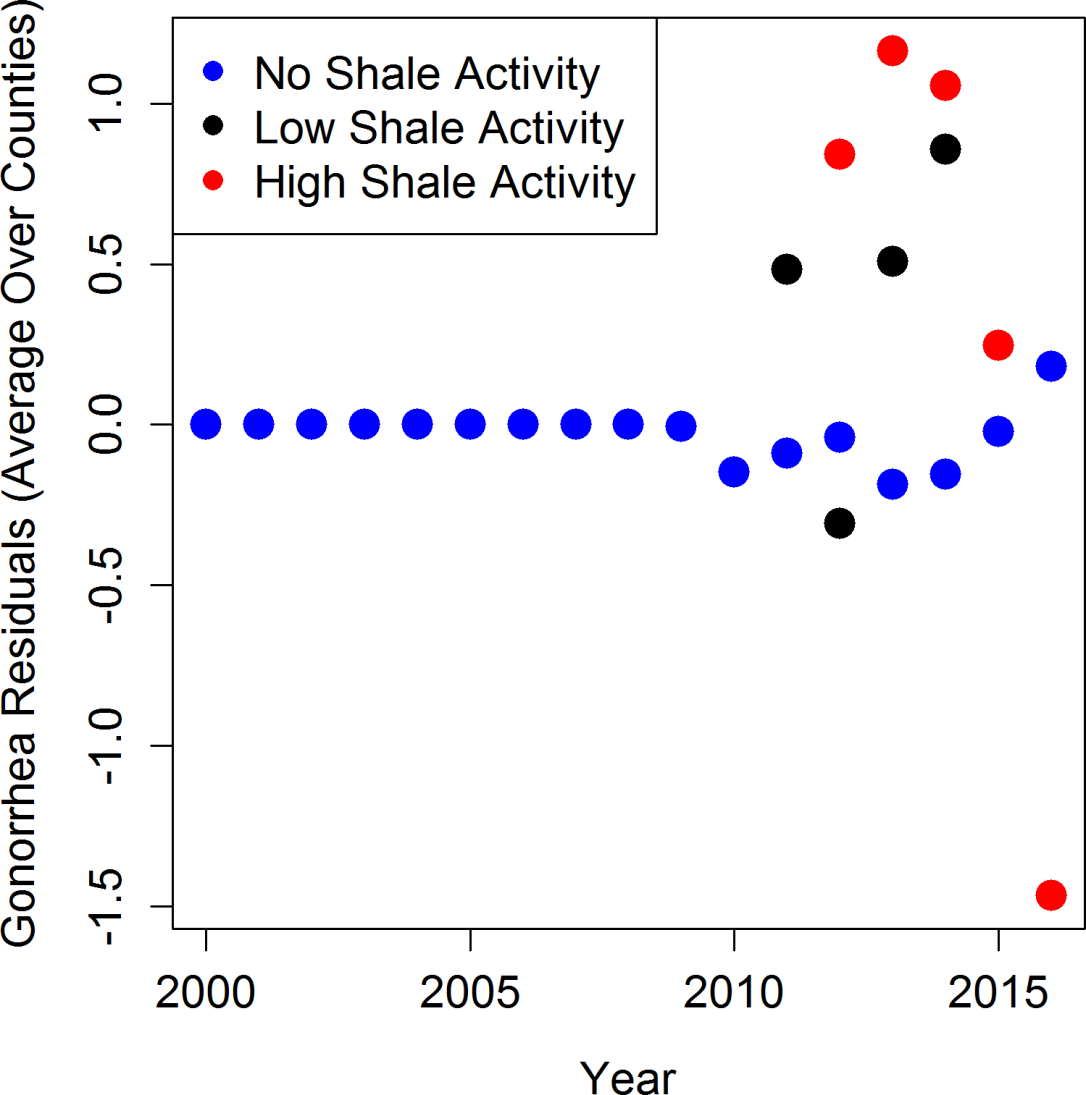
Mean residuals across counties and time by activity categories for gonorrhea models excluding activity variable.

**Fig 7:**
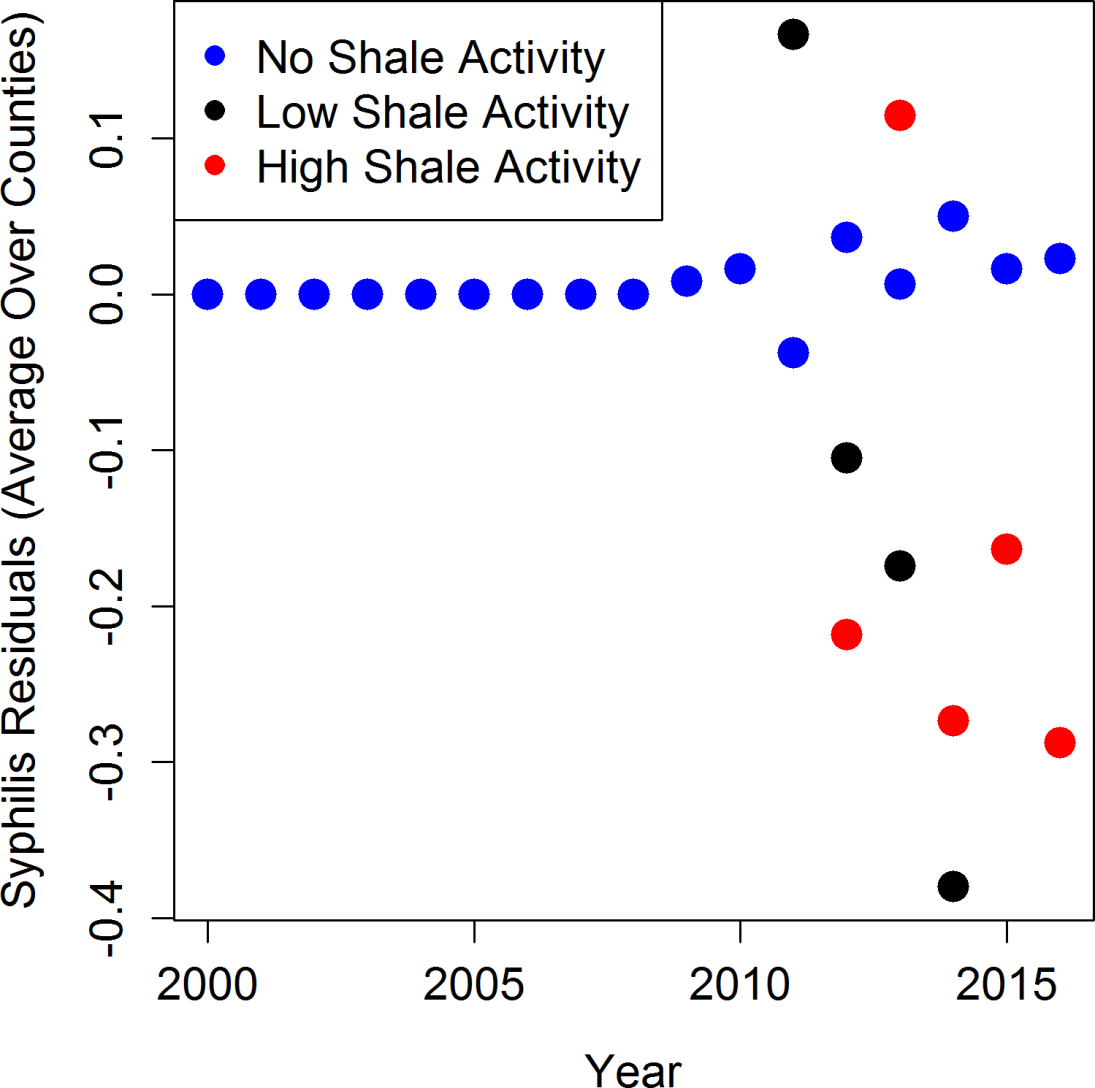
Mean residuals across counties and time by activity categories for syphilis models excluding activity variable.

Mean residuals potentially attributable to shale gas activity are presented in Figs 5, 6 and 7. For each STI across all years, the counties with no shale activity had mean residuals near zero (blue dots), indicating that the model with only sociodemographic covariates provided good fit to the data and there was little unexplained variability. For chlamydia and gonorrhea, the counties that had low (black dots) and high (red dots) shale activity had mean residuals that were often larger than zero, indicating the presence of additional variability in the STI counts not explained by the sociodemographic covariates (Figs 5 and 6). Residuals larger than zero also indicate that the statistical models underestimated the STI counts in these counties when the shale activity variable was excluded. For syphilis, the results were mixed and the residuals were much closer to zero overall, indicating limited impact of shale activity (Fig 7). This graphical evidence in Figs 5, 6 and 7 is consistent with our full statistical models, which indicate that the associations with shale gas activity are statistically significant or borderline statistically significant for chlamydia and gonorrhea, but not for syphilis.

Our numerous sensitivity analyses support the relationships observed in our primary models (S1 Table). Associations were similar between males and females, and were similar whether shale gas activity was characterized using the number of new permits or cumulative well permits. Results were consistent when we parameterized year as a continuous versus categorical variable, although incorporating year as a categorical variable yielded a better fit to the data, even after accounting for the greater number of parameters used. No association was observed between shale gas activity and reported rates of the negative outcome control Salmonellosis for the low or high shale gas activity categories, and results were unchanged when we included Salmonellosis as a covariate in our primary models. For our investigation of residual spatial correlation, none of the hypothesis tests resulted in a statistically significant Geary’s C test statistic (mean *P*-value across all tests = 0.37) after the Bonferroni correction, indicating that the residuals are not spatially correlated and that the inclusion of the county-level random effects, along with the spatially-temporally varying covariates, have adequately removed any residual spatial correlation in the data.

## Discussion

Studies of the potential public health impacts of hydraulic fracturing are growing, and debates continue about the economic, environmental, and human health implications of this industry. Our motivation for this analysis was to advance understanding of the social (i.e., non-chemical) stressors posed by shale gas drilling, including the potential impact of socially-mediated effects of shale gas development on communities and STI transmission [21]. We found that counties with high shale gas activity experienced 21% higher annual rates of chlamydia and 19% higher annual rates of gonorrhea, compared to counties with no shale gas activity, after adjustment for secular trends and other confounders.

Chlamydia and gonorrhea are the two most common notifiable infectious diseases in the US, and these infections are associated with substantial morbidity (including reproductive health consequences) and health care costs [36]. Our findings add to the only other report on this topic that focused solely on gonorrhea [37]. We found a similar magnitude of effect for the association with gonorrhea as in this prior analysis, adding strength to observed associations. The relationship between shale gas activity and increased rates of gonorrhea is particularly concerning because of the recent rise in antibiotic resistant infections. Importantly, we also report the first association with chlamydia, which is far more common than gonorrhea (nearly four times more prevalent in the US), and is the leading preventable cause of tubal factor infertility [28]. Though chlamydia detection is highly influenced by screening practices, our observed magnitude of effect, similar to that for gonorrhea, further strengthens the argument for a true association. We also examined a third STI, syphilis, and observed no association with shale gas activity. In recent years, a substantial majority of cases of syphilis in the US have occurred among men who have sex with men [27]. If most of the increased STI risk in communities associated with shale gas development is due to heterosexual transmission, then we would not expect to see a link with syphilis in this ecologic analysis due to the relatively small proportion of the male population that has sex with other men (<4%) [38]. Our sex-stratified analyses indicate that STI rates increase with shale gas activity among both males and females. This is consistent with our hypothesis that shale gas development may be viewed as a social determinant of health that not only affects the male workers themselves but also has a broader impact on communities. Community impacts were also assessed by Kearney and Wilson (2017), who observed an increase in both marital and non-marital births in communities with shale gas production [39].

It is important to note that in this ecological analysis, we observed associations at the population, not individual, level. It is possible that the reported cases in our dataset do not include the shale gas drilling workers themselves, as they are transient and may be tested and treated (and thus reported) outside of the Ohio counties where they temporarily reside. However, these findings support the idea that the population-level STI rates may be increased through the amplification of sexual mixing patterns that is often associated with migratory workers and is known to increase STI transmission as discussed above [13, 23]. This community-level impact is significant for implications regarding ongoing transmission. In this study, we observed that the STI rates continued to increase at the end of the study period when the number of new permits declined. It is possible that once the STI prevalence has increased in the local community, ongoing transmission may occur in the absence of male workers. Future studies of the shale gas workers themselves or sexual network studies in the communities could provide more insight into this phenomenon.

Although additional research into these associations is warranted, the quantitative evidence provided by our study may be useful in guiding local public health officials and policy makers. Numerous interventions for STI prevention at the individual, group, clinic and population levels have been evaluated and involve behavior change education and counseling, preventative or curative treatments, increased screening, and policy or other environmental changes [40, 41]. Though results have varied, there is strong evidence to suggest that many interventions are effective in reducing risky sexual behaviors and/or STI incidence. In the context of increased shale gas activity, local health departments and health care providers may consider implementing programs such as providing increased access to screening, treatment, and partner services to prevent ongoing STI transmission in their communities. Collaboration with the shale gas industry in these activities should also be considered.

A main strength of this study is the inclusion of multiple STI outcomes. Collectively, the results from these three STI indicate a plausible causal mechanism because of the observed associations for chlamydia and gonorrhea, which were expected, but not for syphilis that is currently concentrated among men who have sex with men. Furthermore, we augment the only previous recent report on this topic that included data from 2003–2013 by incorporating data from 2000 through 2016, the most recent year available. Data were drawn from state, not county records, safeguarding them from any differences in reporting by county. We also used time-varying census data to adjust for multiple potential confounders to minimize threats to the internal validity of the model and analyzed longitudinal data across pre-drilling and post-drilling years to account for secular trends. Further, consistent findings across multiple sensitivity analyses and use of a negative outcome control bolster our results.

The county-level analysis also poses some limitations. Effects of shale gas activity may occur at a more local level, or may spill over into neighboring counties. Our analysis was limited to the state of Ohio, a moderately densely populated, low to moderately diverse population. Associations between shale gas activity and STI rates may differ in states with more urbanized drilling regions, such as in the Barnett shale of Texas or the Denver Julesburg Basin of Colorado. Use of permitted wells could result in some exposure misclassification because some wells (<20%) did not advance to the drilling stage. However, we still consider permit date to be a useful indicator because even among wells that do not get drilled, this phase may involve an influx of workers, surveyors, contractors, and agents. Also, we calculated a metric restricted only to wells in the drilling or producing phase, and it was highly correlated with the metric based on permitted wells (*r*_*Spearman*_ = 0.94). We did not directly adjust for spatial correlation. However, analysis using Geary’s C suggests our approach of including county-level random effects and spatially-temporally varying covariates, accounted for the majority of residual spatial correlation in the data. Other limitations are associated with the outcome of STI case reports that rely on a passive surveillance system. Reported cases are likely underestimates of the true burden of disease, and this may bias our results in an unknown way. Furthermore, given the relative rarity of syphilis infections, our statistical power may have been limited for this outcome. Finally, our annual rates refer to diagnosed and reported cases of STI and may not reflect incident infections.

Whether the association between shale gas development and increased STI rates is causal awaits further investigation. We selected an ecological analysis because we were interested in the population level impact of shale gas drilling; however, future research could explore these trends in individual community members and workers to gain an understanding of the impacts occurring at the individual level. In addition, an analysis into the migratory patterns and lifestyles of migrant workers, as well as the environments and attitudes present in work camps and nearby communities, would be useful in providing a better understanding of how and why such increases in STI rates might occur. Future research would also benefit from analyzing this phenomenon over multiple geographic shale regions to provide robustness to our results.

## Conclusions

STIs are common infections with large individual and community burdens. Our results are the first to our knowledge to quantify the association between shale gas activity and annual rates of multiple STIs. These findings offer insight into the social and health implications which unconventional resource extraction can have on society and warrant further investigation into these issues in other geographic regions.

## Supporting information

S1 Table Rate ratios (RR) and 95% confidence intervals (95% CI) for the association between shale gas activity and reported rates of sexually transmitted infections in Ohio 2000–2016, for various sensitivity analyses. (DOCX)
