# Supplementary material for: Shale gas activity and increased rates of sexually transmitted infections in Ohio, 2000–2016
Source: PLoS One. 2018 Mar 23;13(3):e0194203. doi: 10.1371/journal.pone.0194203 (PMC5865738; doi:10.1371/journal.pone.0194203)
Supplement: S1 Table — (DOCX) [file pone.0194203.s001.docx]

| S1 Table. Rate ratios (RR) and 95% confidence intervals (95% CI) for the association between shale gas activity and reported rates of sexually transmitted infections in Ohio 2000-2016, for various sensitivity analyses. | | | | | | |
| --- | --- | --- | --- | --- | --- | --- |
|  | Original Model^a^ | Male^b^ | Female^b^ | Cumulative Well Activity Metric^a^ | Year as Continuous Variable^c^ | Including Salmonellosis as Negative Control^a^ |
|  | RR (95% CI) | RR (95% CI) | RR (95% CI) | RR (95% CI) | RR (95% CI) |  |
| Chlamydia |  |  |  |  |  |  |
| Low Activity | 0.99 (0.91, 1.08) | 1.04 (0.94, 1.16) | 0.99 (0.90, 1.08) | 1.15 (1.06, 1.25) | 1.07 (0.98, 1.17) | 0.99 (0.91, 1.08) |
| High Activity | 1.21 (1.08, 1.36) | 1.31 (1.13, 1.51) | 1.16 (1.04, 1.31) | 1.09 (1.00, 1.18) | 1.29 (1.15, 1.44) | 1.21 (1.08, 1.35) |
| Gonorrhea |  |  |  |  |  |  |
| Low Activity | 1.14 (0.98, 1.31) | 1.13 (0.96, 1.33) | 1.16 (1.00, 1.35) | 1.24 (1.08, 1.42) | 1.11 (0.97, 1.29) | 1.13 (0.98, 1.31) |
| High Activity | 1.19 (0.98, 1.44) | 1.15 (0.91, 1.44) | 1.21 (0.98, 1.49) | 1.20 (1.05, 1.38) | 1.24 (1.02, 1.51) | 1.17 (0.97, 1.42) |
| Syphilis |  |  |  |  |  |  |
| Low Activity | 0.86 (0.62, 1.20) | 0.94 (0.68, 1.28) | 0.61 (0.34, 1.10) | 0.84 (0.61, 1.16) | 0.76 (0.55, 1.05) | 0.73 (0.52, 1.00) |
| High Activity | 0.71 (0.44, 1.16) | 0.77 (0.48, 1.22) | 0.56 (0.23, 1.37) | 0.69 (0.51, 0.93) | 0.68 (0.42, 1.10) | 0.73 (0.45, 1.18) |
| ^a^Adjusted for population density, median household income, % female, % with health insurance, % White, % Hispanic, % Population 15-29 years old, year (categorical) | | | | | | |
| ^b^Adjusted for population density, median household income, % with health insurance, % White, % Hispanic, % Population 15-29 years old, year (categorical) | | | | | |  |
| ^c^Adjusted for population density, median household income, % female % with health insurance, % White, % Hispanic, % Population 15-29 years old, year (continuous) | | | | | | |
| ^d^ Adjusted for population density, median household income, % female, % with health insurance, % White, % Hispanic, % Population 15-29 years old, year (categorical), and Salmonellosis counts | | | | | | |
